# Persistent Tic Disorders Are Associated With 17q12 Duplications

**DOI:** 10.21203/rs.3.rs-7031850/v1

**Published:** 2025-08-19

**Authors:** Matthew Halvorsen, Sheng Wang, Tyne Miller-Fleming, Dongmei Yu, Apostolia Topaloudi, Elles de Schipper, Julia Bäckman, David Mataix-Cols, Christian Rück, Behrang Mahjani, Joseph Buxbaum, Dorothy Grice, Larisa Cavallari, Dominick Angiolillo, Francesco Franchi, Lea Davis, Lide Han, Douglas Ruderfer, Andrea Dietrich, Pieter Hoekstra, Manuel Mattheisen, Luz Porras, Paola Giusti-Rodríguez, Carol Mathews, Peristera Paschou, Jeremiah Scharf, Jeremy Willsey, James Crowley

**Affiliations:** University of North Carolina at Chapel Hill; Massachusetts General Hospital; Karolinska Institutet; Karolinska Institutet; Icahn School of Medicine at Mount Sinai; Mount Sinai School of Medicine; Icahn School of Medicine at Mount Sinai; Vanderbilt University Medical Center; Dalhousie University; University of Florida College of Medicine; Purdue University; UNC Chapel Hill

## Abstract

Tourette Syndrome (TS) and Persistent Tic Disorder (PTD) are childhood-onset neuropsychiatric conditions with high heritability. Due to current sample size limitations, identifying TS/PTD risk genes has been challenging. This study addressed this issue by conducting a meta-analysis of microarray copy number variant (CNV) studies from three TS/PTD genomics consortia, supplemented with new data from 3,291 cases. This approach more than doubled the sample size of previous TS/PTD CNV studies, with CNV calls generated from 5,725 TS/PTD cases and 10,982 matched controls. The results confirmed that TS/PTD cases 1) have a higher burden of ultra-rare deletions overlapping loss-of-function intolerant genes (OR = 1.68, P = 9.3×10^−5) and 2) are more likely to carry established neurodevelopmental CNVs (OR = 1.42, P = 3.9×10^−2) compared to controls. Additionally, a novel, genome-wide significant CNV locus for TS/PTD was discovered, involving duplications at 17q12 (hg19 chr17:34.8 – 36.2 Mb). This locus is associated with a known duplication syndrome associated with variable neuropsychiatric traits, but has not been previously linked to tic disorders. Eight cases and one control carried the canonical ~1.4 Mb duplication at chr17:34.8 – 36.2 Mb, while one additional case had a smaller 110 kb duplication within this known CNV that included only one gene, *ACACA* (acetyl-CoA carboxylase, OR = 26.7, P = 5.69×10^−7). Overall, this study provides further evidence that rare, genic CNVs play a substantial role in the genetic architecture of TS/PTD and identifies a new genome-wide significant association with this neurodevelopmental disorder.

## INTRODUCTION

Tic disorders are a spectrum of developmental neuropsychiatric conditions characterized by childhood-onset, repetitive, stereotyped, and partially suppressible muscle movements and/or vocalizations.[1] These disorders can cause significant emotional and physical distress, as well as impairment for patients and their families, and, in some cases, may lead to lifelong disability and an increased risk for suicide.[2] The two most common and closely related tic disorders, Tourette Syndrome (TS) and Persistent Tic Disorder (PTD), are defined by the presence of motor and/or vocal tics that onset in childhood or adolescence and persist for one year or more. TS/PTD has a combined prevalence of approximately 2% among children aged 5 to 14, with the prevalence of TS in this age range estimated at 0.5–0.8%.[3, 4] Despite their high prevalence and functional consequences, tic disorders remain understudied compared to other neuropsychiatric conditions.

TS/PTD is highly heritable (~ 60–70%) based on twin[5] and family[6] studies. First-degree relatives of affected individuals have an approximately 20-fold increased risk of TS/PTD compared to the general population[6], representing one of the highest recurrence risks for a neuropsychiatric disorder. In line with these epidemiological findings, a recent TS genome-wide association study (GWAS) estimated that common genetic variation contributes 0.21 to overall heritability[7], and larger studies are currently underway. Two moderate-sized whole exome sequencing (WES) studies have been published, revealing a significant burden of *de novo* gene-damaging coding variants in TS probands, and six high-confidence TS risk genes have been identified thus far[8, 9]. Rare or *de novo* copy number variants (CNVs) also play a role in the risk of TS/PTD. The largest CNV study to date examined a cohort of 2,434 TS cases and 4,093 unaffected controls, finding that cases were more likely to carry rare CNVs that disproportionately affected protein-coding genes[10]. This study also identified two additional TS risk genes: *CNTN6* and the established neurodevelopmental gene *NRXN1*.

**Figure 1:**
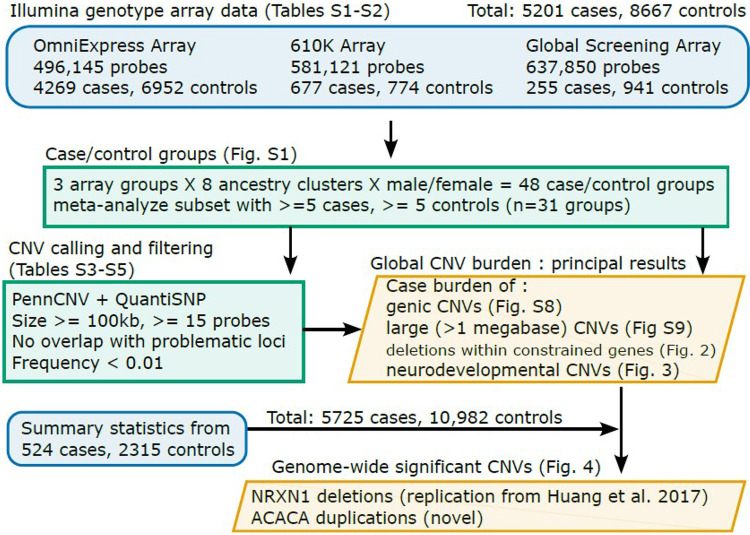
Overview of this TS/PTD CNV meta-analysis and organization of figures and tables. Blue indicates the raw data used, green represents details of the analysis, and orange lists key results.

The current study enhanced statistical power for detecting novel TS/PTD CNVs and risk genes by combining new genotype array data from 3,291 cases with all available existing TS/PTD array data from three TS/PTD genetics consortia: the Tourette Association of America International Consortium for Genetics (TAAICG),[10] the Tourette International Collaborative Genetics study (TIC Genetics),[11] and the European Multicentre Tics in Children Study (EMTICS).[12] We performed a meta-analysis of these datasets, generated across various genotyping array types, by consistently re-calling all CNVs from harmonized groups of array data. After extensive quality control, the total sample size included 5,725 cases and 10,982 controls, which is more than double the sample size of the largest published TS/PTD CNV study to date. Our analyses included a global CNV burden analysis and gene and locus-based association tests. Figure 1 provides an overview of the steps taken in this meta-analysis, in addition to indicating which tables and figures correspond to each step.

## METHODS

### Cases and controls

#### Informed consent

was obtained from all participants, and ethics board approval was secured from all institutions before starting this study. Details about the sample, including clinical characterization, inclusion criteria, and genotyping, can be found in the **Supplemental Methods**.

### Raw data

Raw data were obtained from 17 genotype array datasets (**Table S1**). For 16 of the 17 datasets, the data were received as IDAT files, from which we utilized the provided cluster and project file(s) to harmonize the data and subsequently to call CNVs. For the 17th dataset, we received CNV calls themselves. Further details regarding raw data received, along with additional quality control (QC) before CNV calling, can be found in the **Supplemental Methods** section.

### Formation of array groups before CNV calling

We pruned single nucleotide polymorphisms (SNPs) and samples with high missingness from each dataset using PLINK v1.90b6.24. We excluded all SNPs from the 17 input datasets with a missingness rate greater than 0.02 and all samples with genotype missingness exceeding 0.02. We then merged the pruned datasets genotyped on the same microarray platform (610K, GSA, and OmniExpress), retaining overlapping SNPs with a missingness rate of ≤ 0.02 and samples with genotype missingness of ≤ 0.02 corresponding to these qualifying SNPs. The genotype data were merged using SNP IDs from all datasets for a given platform, excluding SNPs with discordant genomic coordinates. We formed three distinct ‘groups’ of genotype array data (**Table S1**): 1) TS GWAS Illumina 610K cases and controls, along with ALZ 610K controls, 2) Illumina GSA TS cases, GSA controls, and GSA GPC control samples, and 3) Illumina OmniExpress, OmniExpressExome, and Duo 1.2M array datasets. **Table S2** lists the number of samples per dataset after each QC step, while **Tables S3** and **S4** present the deletion and duplication call rates per dataset after each CNV filtering step. **Table S5** illustrates the array group case/control call rates following each CNV filtering step.

### CNV calling on sample-level data

CNV calling was performed on the set of SNPs used to merge hard call genotypes within each of the three array groups. Consequently, each array group had a different number of probes available for CNV calling. The OmniExpress group, which had the most samples, also had the fewest probes, totaling 496,145. The GSA and 610K groups featured 637,850 and 581,121 probes for calling, respectively. We defined a set of 61,253 SNPs shared across all 3 dataset groups and used these SNP genotypes for further assessments of relatedness and genetic similarity (see Supplemental Methods for how these SNPs were derived). We followed the CNV calling procedure described by Huang et al.[10]. See **Supplemental Methods** for details.

### Clustering according to genetic ancestry

After controlling for cryptic relatedness between samples (see **Supplemental Methods**), we applied Louvain clustering (details in **Supplemental Methods**) on principal component analysis (PCA) results of common variant genotypes to create sample clusters independent of case status and specific ancestry, as in other rare variant studies [13, 14]. We conducted PCA on the LD-pruned genotype matrix (**Figure S1**), using PCs 1–6 for clustering. PCs 1–6 captured most of the PCA variance (Scree plot, **Figure S2**) and were primarily influenced by ancestry differences (**Figure S3**), thus being used for clustering. We identified eight distinct clusters based on ancestry (**Figure S4**). The first three clusters contained most of the dataset and appeared to correspond to individuals of European ancestry. It is important to note that approximately 20% of cases are categorized into one of the other clusters, and this method allows us to include these individuals in the analysis.

### Sample-level outlier removal and defining qualifying CNVs

We used intensity metrics generated by PennCNV (log R ratio standard deviation [LRR SD], B allele frequency [BAF] drift, GCWF) to identify samples classified as outliers compared to others (more than three standard deviations from the median on any of these metrics). This process was carried out for each dataset, and samples were removed if any of these three metrics were classified as outliers compared to others.

Next, we applied CNV-based QC thresholds to array groups, focusing on the raw CNV count and the number of bases occupied by raw CNV calls per sample. For the OmniExpress group samples, we determined that there should be no more than 15 raw CNV calls in each sample, summing to no more than 20 Mb. For the 610K and GSA samples, we stipulated a maximum of 30 raw calls, totaling no more than 20 Mb. These methods standardized the raw CNV call rates across the datasets, although the QC metrics varied among the three array groups (**Figure S5**). The LRR SD metrics per dataset were less consistent than the raw CNV call rates, which aligned with the heterogeneous quality of the input data and supported concentrating the analysis on larger CNVs less susceptible to noise (**Figures S6-S7**).

Among the raw CNV calls in sample-level data, we considered CNVs for analysis if they met the following criteria: spanning at least ten probes; length between 30kb and 20Mb; overlapping chromosomes 1–22. For more details on filtering qualifying CNVs and calibrating CNV call thresholds for analysis, see the **Supplemental Methods**.

### Controlling for differences in array group, ancestry, and sex among cases and controls

We included 13,869 samples (5,202 cases and 8,667 controls) with sample-level CNV call data for global analyses of CNV burden. We stratified these samples into 31 case/control groups, each representing a unique combination of array group, ancestry cluster, and sex. Each case/control group was required to have at least five cases and five controls, with the fraction of cases ranging from 0.10 to 0.90. The creation of these groups allowed us to compare case/control CNV burden using a regression framework, along with a complementary set of two-sided Cochran-Mantel-Haenszel tests employing 2×2 contingency tables showing the number of cases and controls with at least one qualified CNV and the number without any qualified CNV.

### Adapting the prior regression framework to the full case/control dataset

To adapt the regression framework for meta-analysis across multiple ancestries and array groups, we modified the model with a series of adjustments. First, instead of using binary indicators for sex and ancestry cluster membership, we switched to the label ‘GROUP’, denoting the unique combination of array group, ancestry cluster, and sex to which each sample belonged. As proof of principle, we reanalyzed the replication cohort from Huang et al. utilizing this modified model, yielding results similar to the original findings (for details, see Supplemental Methods). Since the number of raw CNV calls significantly differed between cases and controls despite accounting for array group, ancestry, sex, and LRR SD differences, we included the number of raw calls as a covariate in the complete analyses. Our logistic regression model thus became:

### TS/PTD_status ~ Burden_metric + GROUP + LRR_SD + n_cnv_raw

We then used this revised model to replicate the analyses of the Huang *et al*. study. We compared CNV counts in TS/PTD cases and controls genotyped on the OmniExpress array group and confirmed an elevated CNV burden in cases, as in the original study. The same patterns of CNV enrichment were noted in TS/PTD case samples relative to controls in the Illumina 610K and GSA sample groups.

### Defining test loci for genome-wide analyses of CNV burden in TS/PTD cases and control groups

We defined two sets of genomic loci for CNV meta-analysis across case/control groups stratified by array group, ancestry, and sex. The first set of loci corresponded to coding regions from CCDS transcripts within Ensembl GRCh37.87 and was used for explicit gene-based deletion or duplication burden tests. The second set was a sliding window of 200kb in size, advancing in 10kb steps, and employed for tests of deletion or duplication burden in annotation-agnostic regions of the genome. These sets of loci were stored in BED files and used to create case/control count data for global CNV burden tests. We also applied this approach to external datasets for which we could not directly obtain sample-level calls due to restrictions on individual-level data sharing (see below).

### External CNV call sets

We also received three additional, independent, unpublished datasets of CNV call summary statistics (i.e., not sample-level data) from matched TS/PTD cases and unaffected controls: the Nordic OCD and Related Disorders Consortium (NORDiC), Epidemiology and Genetics of Obsessive-compulsive disorder and chronic tic disorders in Sweden (EGOS), and the Vanderbilt DNA Biobank (BioVU). Specific details about the generation of these CNV call sets and the filtering procedures implemented for each dataset are as follows (for more details, see **Supplemental Methods**):

#### NORDiC Swedish TS/PTD cases and controls:

The NORDiC study has been detailed previously.[15] CNV calls were extracted from 188 TS cases and 414 unaffected controls (all unrelated and of European ancestry). These data had already undergone quality control (see **Supplemental Methods**), and the CNV calling and filtering process was consistent with what was described above. We created count tables as outlined previously, dividing the case/control data into two groups: a male-only group (112 cases, 199 controls) and a female-only group (76 cases, 215 controls).

#### EGOS Swedish TS/PTD cases and controls:

The EGOS study has been previously described.[16] A total of 174 unrelated European-ancestry TS/PTD cases and 1,123 population-matched unaffected controls were selected for analysis. Samples underwent prior quality control (see Supplemental Methods), and all had definable data-derived sex. CNV calls were made using the CNVision pipeline, which integrates CNV calls made with PennCNV, QuantiSNP, and GNOSIS. We required that, within the CNVision output, a CNV call be supported by both PennCNV and QuantiSNP to be included in the analyses. As with the NORDiC case/control data, CNV filtering protocols were consistent with those described above.

#### BioVU TS/PTD cases and controls:

The BioVU project has been previously described.[17] A total of 93,626 patients from the Vanderbilt BioVU biobank were genotyped using the Illumina Multi-Ethnic Genotyping Array (MEGA EX). To enhance the quality of input for CNV calling, the total variants (n = 2,038,233 SNPs) were limited to those with high genotyping call rates (> 95%). CNVs were identified as described in **Supplemental Methods**, employing PennCNV along with a PFB file and GC model file created from 1,200 randomly selected samples in the biobank.[18] Samples with a LRR SD < 0.3, BAF drift < 0.01, and an absolute waviness factor (absWF) < 0.05 were retained. Only CNVs exceeding 10 kb and containing at least 10 contributing variants were retained. Samples with outlier counts (absolute values of z-scores greater than 1.96) of CNVs after quantile normalization were also excluded. CNVs were discarded if they overlapped with genomic regions such as centromeres, telomeres, and ENCODE blacklist areas.[19] Adjacent CNVs were merged if the intervening gap was less than 20% of the total length of the merged CNV. Finally, only CNVs present in less than 1% of the samples (allele frequency < 0.5%) were kept for analysis. A total of 945,196 CNVs among 86,294 samples were retained following the quality control procedures.

We conducted sample-level QC to exclude individuals with anomalous CNV call data and selected suitable TS/PTD cases and unaffected controls. We first excluded individuals with more than 50 CNV calls, CNV calls longer than 20 Mb, or individuals whose DNA fidelity might be compromised (e.g., due to blood transfusions or blood cancers), to align with the pipeline for internal datasets. We then applied a previously defined diagnostic algorithm[20] to identify 303 TS/PTD cases (178 males and 125 females) and 1,531 age and sex-matched controls (806 males and 725 females) to form a case/control dataset. These samples were further stratified by sex, and bedtools[21] was utilized to obtain the count of deletion/duplication calls per case/control group for previously defined genomic windows and protein-coding gene loci. The count tables were subsequently employed for meta-analyses.

### Association testing at both the gene and locus levels

We combined the callsets with rare CNV counts for datasets where we did not have access to individual-level data from the BioVU (N = 1,834, comprising 303 cases and 1,531 controls), EGOS (N = 1,297, including 174 cases and 1,123 controls), and NORDiC (N = 602, consisting of 188 cases and 414 controls) projects into a single dataset to enhance our capacity to identify risk loci. Aggregated counts by gene or locus were provided by our collaborators on these studies using the same pipeline. However, due to our lack of access to individual-level data, we could not perform BAF filtration, as we did with the dataset with available individual-level data. We conducted two rounds of association tests to identify significantly associated CNV loci and bolster confidence in these findings. First, we generated raw results using all CNV calls from these datasets. Our collaborators from each group (NORDiC, EGOS, BioVU) then visually validated the CNVs associated with the genes of interest. We re-conducted the association test to obtain the final results after excluding the presumably spurious CNV calls that were not manually validated on-site.

For the final analyses of all 20 datasets, we conducted locus-specific association tests at both the gene and breakpoint levels. For the gene association test, we included only CNVs that overlapped coding sequences. For the breakpoint test, since CNVs were derived from microarray platforms with varying SNP distributions, we developed a strategy to scan the genome stepwise (step = 10 kb) with a fixed window (size = 200 kb). Gene- and locus-based association tests were performed separately for deletions and duplications. We generated p-values using the Cochran–Mantel–Haenszel test to control for biases introduced by population stratification, array platforms, and experimental batches. Genome-wide significance thresholds were determined through permutations using the min(P) method. Briefly, we permuted 10,000 times by randomly switching the phenotype labels and calculated p-values for each window. We then recorded the minimum p-value for each iteration. Following permutation, the top 5% quantile point of the minimum p-values was used as the genome-wide significance threshold. Significance thresholds for deletions and duplications were defined independently for gene and breakpoint association tests. Using the permuted p-values, we computed the adjusted p-values controlling for the family-wise error rate (FWER) with the NRejections package. Lastly, we multiplied the p-values by two to account for the two rounds of association tests.

## RESULTS

### Global CNV burden analysis

We compared the burden of CNVs that were > 100kb and encompassed at least 15 probes (see **Supplemental Methods** and **Table S6** for the derivation of thresholds) across 13,869 samples (5,202 cases, 8,667 controls) from the 17 datasets with individual-level call data that passed QC. We did not detect significant evidence of an elevated global burden of these CNVs in cases compared to controls (OR = 1.03, P = 0.22). However, we observed an excess burden of very large (> 1 megabase) CNVs in cases relative to controls, which is consistent with findings previously described in Huang et al. [10] (OR = 1.31, P = 2.16×10−2, **Figure S9**). Although we did not find an excess of singleton CNVs in cases compared to controls (**Figure S10**), we did find that very large CNVs (i.e., > 1 megabase) were more likely to be singletons when identified in a case (OR = 1.60, P = 0.02). Interestingly, even within these very large CNVs alone, we observed that CNV calls in cases were larger than those in controls (P = 0.01). As reported previously, a significant portion of the CNV burden in TS/PTD cases relative to controls in this study appeared to be associated with protein-coding genes [10]. We classified CNVs as genic if they overlapped at least one protein-coding base and observed that, although there was no difference in non-genic CNV burden (OR = 0.96, P = 0.33), there was an increased burden of genic CNVs in cases relative to controls (OR = 1.09, P = 1.59×10−2, **Figure S11**).

**Figure 2:**
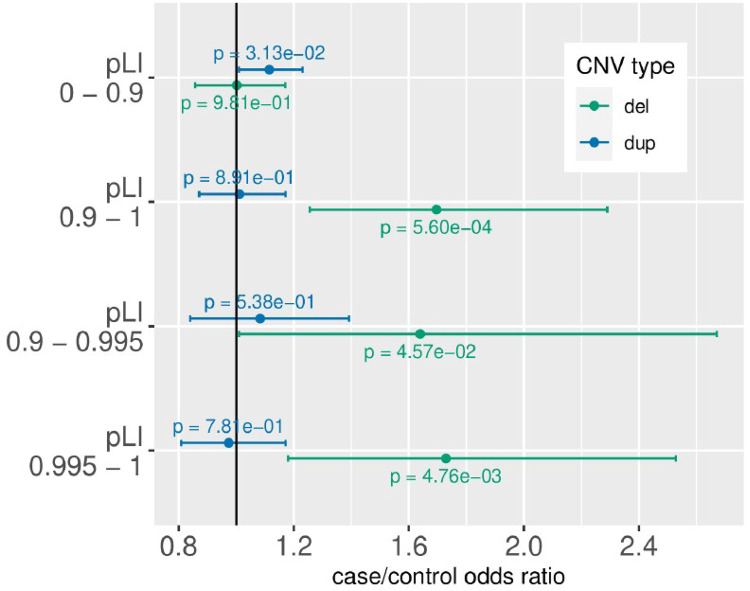
CNV burden stratified by CNV type and genic constraint.

Most of the excess of genic CNVs in TS/PTD cases was linked to deletions specific to genes that were intolerant of lower gene dosage (Fig. 2). We defined this intolerance based on the probability of loss-of-function intolerance (pLI) outlined elsewhere [22], using a binary threshold of pLI > 0.9. Furthermore, we defined a bin of highly intolerant genes with pLI > 0.995 as described in Satterstrom et al. 2020[23] and Fu et al. 2022[24]. We observed a heightened burden of deletions affecting intolerant genes (pLI > 0.9, OR = 1.70, P = 5.60×10−4, Fig. 2), but no excess of deletions in genes tolerant for loss of function mutations (pLI < 0.9, OR = 1.00, P = 0.98) but These findings were consistent with an additional test utilizing genes identified as haploinsufficient from large-scale CNV data (**Figure S12**).

**Figure 3:**
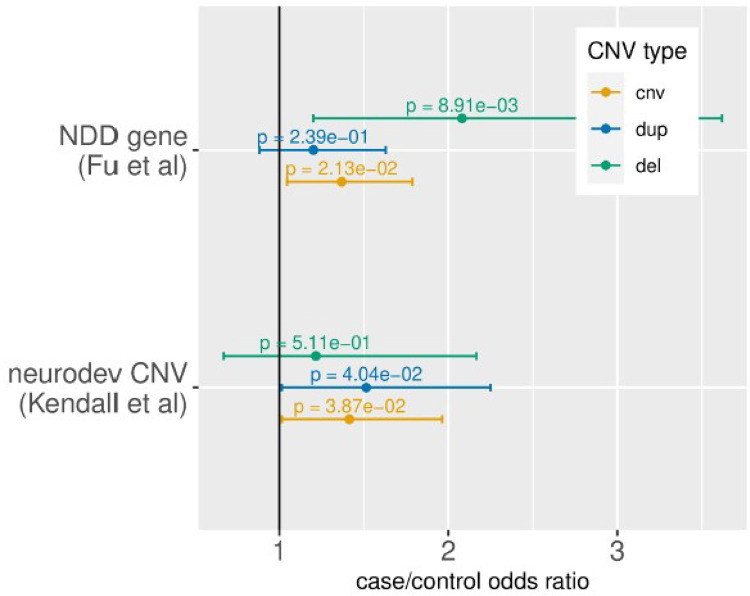
CNV burden in known neurodevelopmental genes and CNVs.

TS/PTD cases were more likely than controls to have CNVs associated with an increased risk for other neurodevelopmental conditions. TS/PTD cases exhibited a higher frequency of CNVs that were manually curated as neurodevelopmental (see Kendall *et al*. [25], OR = 1.42, P = 3.87×10−2), and enriched in CNVs impacting neurodevelopmental risk genes (n = 664) identified in a recent meta-analysis of exome sequencing data (see Fu et al. [24], OR = 1.37, P = 2.13×10−3). This result was mainly driven by deletions (OR = 2.08, P = 8.91×10−3, Fig. 3). Refer to **Table S7** for the case/control groups for these analyses, **Table S8** for the complete results, and **Table S9** for leave-one-out analyses of global CNV burden.

We also evaluated the overlap between the TS/PTD CNV case/control summary statistics and available TS/PTD trio whole exome sequencing data as outlined in Wang et al.,[9] Liu et al.,[26] and Zhao et al.[27] to ascertain whether TS/PTD cases exhibited an increased incidence of CNV deletions in genes affected by *de novo* variation in these studies. We identified a set of 37 genes from these publications that had 1) pLI > 0.5 and 2) at least one *de novo* protein-truncating variant call across the studies, and assessed the case/control CNV deletion burden in our dataset. Despite the low CNV deletion call rate across the data coinciding with one of these genes (0.0001 per control sample), we observed significant enrichment in cases compared to controls (~ 0.001 per case; Fisher’s exact test OR = 8.34, P = 0.03). These findings indicate that TS/PTD risk gene discovery in exome sequencing studies would improve with the addition of CNV call statistics, as demonstrated in Fu et al. for ASD.[24]

### Gene and locus-based association test findings

Next, we conducted gene and locus-based association tests to identify individual TS/PTD risk genes and loci. As mentioned in the methods, we enhanced statistical power by incorporating summary count data from external CNV call sets (665 cases and 3,068 controls) into our individual-level datasets, resulting in a total sample size of 5,867 cases and 11,735 controls (**Table S7**) Due to limited access to post-QC CNV calls for these external datasets, we carefully verified any significant genes or loci through visual inspection. Since there are no established thresholds for genome-wide significance for CNVs, we generated thresholds and adjusted p-values empirically by performing 10,000 label-swapping permutations using the min(P) method (limited to array group X ancestry cluster X sex). We multiplied the adjusted p-values by two to account for two rounds of association tests (one for deletions and one for duplications)(**Tables S10, S11**).

**Figure 4:**
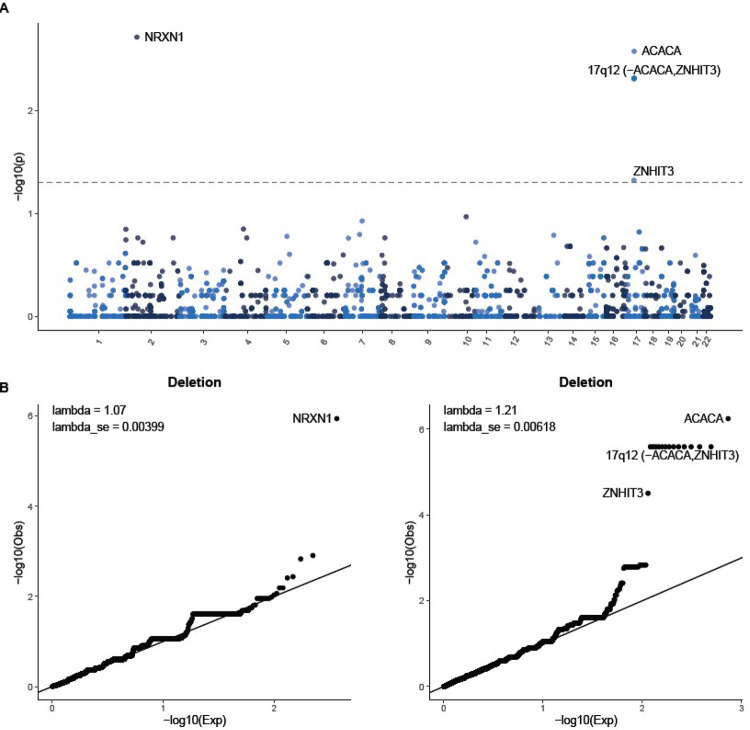
Gene-based CNV associations. A) The association between rare CNV burden (deletions and duplications) and all protein-coding genes. The dotted line indicates the genome-wide significance threshold for adjusted p-values based on permutation using the min(P) method. B) Q-Q plot of gene-based association tests for deletions and duplications. The lambda values near one indicate that the results are well-controlled and no systematic bias exists.

Gene association tests for deletions identified *NRXN1* as significantly associated with TS, with 17 cases and three controls carrying deletions that overlap coding bases (OR = 11.84 [3.20–43.83], P = 1.14E-6, adjusted P = 0.0019, consistent with Huang et al. [10] (see Fig. 4A). This over-representation was further confirmed by breakpoint association analysis (P = 8.76E-6, adjusted P = 0.015). **Table S12** presents the full summary statistics from locus-based association tests. Figure 4B presents a QQ plot of gene-based association tests for deletions and duplications separately. *NRXN1* is the sole significant association, with the genomic inflation factor lambda at 1.06, consistent with the absence of systemic bias, such as population stratification.

Regarding duplications, we did not replicate the significant association between TS and *CNTN6* identified by Huang et al. However, we found a novel TS-associated duplication on 17q12 that was significant in both gene and locus-based tests (see Fig. 4A). This is a gene-rich region with most duplications spanning approximately 1.4 Mb and including 15 genes (hg19 chr17:34.8–36.2 Mb). In addition to the 8 TS cases with the canonical 17q12 duplications, an additional TS case was found to have a small 110kb duplication that only impacts the 17q12 gene *ACACA* (Acetyl-CoA Carboxylase Alpha), leading to a slightly higher significance for this gene than the other 14 genes. Specifically, duplications affecting *ACACA* were observed in nine TS/PTD cases and one control (for breakpoints, see **Figure S12**), corresponding to an OR of 26.72 (3.59–199.00) (P = 5.69E-07, adjusted P = 0.0027 for gene association analysis, P = 2.62E-7, adjusted P = 0.0010 for breakpoint association analysis).

The gene product of *ACACA* is highly expressed in lipogenic tissues such as the brain. *ACACA* expression can be detected in single-cell RNA-seq data from brain cell types in fetal and adult brain, including astrocytes, excitatory neurons, and inhibitory neurons (See Supplementary Methods, **Figure S14**). Integration with genome-wide chromatin conformation capture data (Hi-C) from fetal and adult cortex identified a number of regulatory loops (i.e., involving enhancers and promoters; see **Figure S15**) and frequently interacting regions (FIREs), which are associated with cell-type-specific gene regulation. [29] Using Hi-C data, we also found that the identified duplication that includes the *ACACA* locus overlaps a topologically associated domain (TAD) boundary.

## DISCUSSION

This study enhanced the statistical power to detect novel TS/PTD CNVs and risk genes by merging data from three TS/PTD genetics consortia and supplementing these results with additional CNV case/control summary count data from three biobanks. Since these datasets were generated using different genotyping arrays, we matched cases and controls on the basis of genetic similarity and conducted the analysis within each array group before meta-analyzing the association results. After thorough quality control, our sample included 5,867 cases and 11,735 controls, which more than doubles the size of the largest TS/PTD CNV study conducted to date. Our burden results confirm a previous observation that ultra-rare CNVs overlapping constrained protein-coding genes are associated with TS/PTD risk.[10] Additionally, TS/PTD cases were more likely to have known neurodevelopmental CNVs and CNVs affecting genes previously identified as neurodevelopmental risk genes through exome sequencing.

With this increase in sample size, we detected one novel, genome-wide significant TS/PTD CNV association: duplications at 17q12. The rate of 17q12 duplications in cases (9 of 5,867) surpassed the rate in controls (1 of 11,735) and was significant for both gene and locus-based tests. The implicated region of 17q12 (hg19 chr17:34.8 – 36.2 Mb) contains 15 genes, and recurrent duplications in this area lead to 17q12 duplication syndrome (OMIM # 614526, see ***Figure S12***). Fewer than 100 cases of 17q12 duplication syndrome have been reported, featuring variable levels of intellectual disability, seizures, autism spectrum disorder, schizophrenia, aggression, and self-injury.[28] While tic disorders have not been previously associated with 17q12 duplications, it is unclear if tics were assessed in previous studies. Most 17q12 duplications (~90%) in the literature are inherited from a minimally affected parent, resulting in variable penetrance.[28] In addition, a reciprocal 17q12 deletion syndrome exists that also presents with neuropsychiatric symptoms; however, the majority (~75%) of these deletions occur *de novo* and exhibit higher penetrance than the duplications.[28] It remains unclear which gene(s) impacted by these CNVs may account for the phenotypic features observed in these syndromes.

Notably, we observed a 110 kb duplication in a TS/PTD case affecting only the gene *ACACA* (Acetyl-CoA Carboxylase Alpha) within the 17q12 region. This gene is involved in fatty acid synthesis and has not been previously associated with neurological or psychiatric conditions, but has broad expression in the fetal and adult brain. As noted above, using Hi-C data, we found that the 110 kb duplication that includes the *ACACA* locus overlaps a topologically associated domain (TAD) boundary. This is notable because deletion or duplication of this TAD could disrupt interactions between regulatory elements located in neighboring TADs and their target promoters, and could lead to altered gene expression.

This study’s other genome-wide significant finding involved deletions impacting the coding bases of *NRXN1* (17 in cases, 3 in controls). *NRXN1* deletions are associated with multiple neuropsychiatric and developmental disorders and have been previously linked to TS/PTD by Huang et al.[10] We observed only weak evidence to support Huang et al.’s other identified significant association between TS/PTD and duplications of *CNTN6* (28 in cases, 19 in controls, nominal p = 0.039, adjusted p = 0.44). However, this may be due to the inclusion of various genotyping array types, which have differing levels of probe coverage over *CNTN6*, or the possibility that the CNTN6 duplication is population-specific. Overall, our burden results suggest that CNVs at many different loci influence TS/PTD risk, but our current sample size is insufficient to detect all of them. These results are consistent with CNV studies of other neuropsychiatric conditions, such as schizophrenia[30] and attention deficit hyperactivity disorder [31], at similar sample sizes.

This study has some limitations. First, as noted above, this meta-analysis is restricted to genotype array data across different platforms. Consequently, probe coverage and our ability to detect CNVs at specific loci varied among cohorts. Second, we confined our CNV calls to those at least 100 kb in size due to limited probe density and our aim to prioritize reliable CNV calls. Third, approximately 80% of the samples in this study are from individuals of European ancestry. Therefore, the representation of global populations is constrained.

In conclusion, we have confirmed that rare, genic CNVs significantly contribute to the genetic architecture of TS/PTD, replicated a previously reported association, and identified a novel, genome-wide significant association. There are likely many genes for which dosage influences TS/PTD risk, aligning with findings from GWAS and WES studies of TS/PTD. Future research will benefit from employing higher-resolution methods, such as WES and whole genome sequencing, to investigate CNVs in larger and more diverse samples. Ultimately, a comprehensive analysis of rare CNVs, SNVs, and indels in a significantly increased sample will uncover a core set of TS/PTD risk genes.

## CONSORTIA

### Psychiatric Genomics Consortium Tourette Syndrome Working Group (PGC-TS)

Thomas D. Als, Harald Aschauer, Gil Atzmon, Matie Bækvad-Hansen, Valentina Baglioni, Csaba Barta, Cathy L. Barr, Nir Barzilai, James R. Batterson, Robert Batterson, Noa Benaroya-Milshtein, Fortu Benarroch, Cheston Berlin, Julia Boberg,, Bianka Burger, Anders D. Børglum, Lawrence W. Brown, Ruth Bruun, Cathy L. Budman, Randy L. Buckner, Joseph D. Buxbaum, Jonas Bybjerg-Grauholm, Francesco Cardona, Danielle C. Cath, Keun-Ah Cheon, Sylvain Chouinard, Barbara J. Coffey, Giovanni Coppola, James J. Crowley, Niklas Dahl, Lea K. Davis, Sabrina M. Darrow, Mark J. Daly, Christel Depienne, Silvia De Rubeis, Andrea Dietrich, Yves Dion, Diana R. Djurfeldt, Laura Domenech-Salgado, Valsamma Eapen, Erik Elster, Dana Feldman, Thomas V. Fernandez, Nelson B. Freimer, Carolin Fremer, Blanca Garcia-Delgar, Marcos Madruga-Garrido, Donald L. Gilbert, Paola Giusti-Rodriguez, Marco Grados, Erica Greenberg, Jakob Grove, Dorothy E. Grice, Matt Halvorsen, Andreas Hartmann, Bjarne Hansen, Jan Haavik, Julie Hagstrøm, Johannes Hebebrand, Tammy Hedderly, Gary A. Heiman, Luis Herrera, Isobel Heyman, Anke Hinney, Matthew E. Hirschtritt, Pieter J. Hoekstra, Jae Hoon Sul, Hyun Ju Hong, David M. Hougaard, Alden Y. Huang, Chaim Huijser, Franjo Ivankovic, Joseph Jankovic, Christina Kappler Friedrichs, Elinor K. Karlsson, Jakko A. Kaprio, Young Key Kim, Young-Shin Kim, Robert A. King, Nadine Kirchen, Carolin-Sophie Klages, James A. Knowles, Yun-Joo Koh, Sodahm Kook, Najah Khalifa, Anastasios Konstantinidis, Christoph Kraus, Samuel Kuperman, Roger Kurlan, Gerd Kvale, James Leckman, Paul C. Lee, Bennett Leventhal, Holan Liang, Paul Lichtenstein, Kerstin Lindbald-Toh, Thomas Lowe, Pétur Luðvigsson, Jurjen J. Luykx, Gholson J. Lyon, Behrang Mahjani, Osman Malik, David Mataix-Cols, Manuel Mattheisen, Carol A. Mathews, Irene A. Malaty, Davide Martino, William M. McMahon, Andrew McQuillin, Sandra M. Meier, Marieke D Messchendorp, Tyne Miller-Fleming, Pablo Mir, Rainald Moessner, Astrid Morer, Preben B. Mortensen, Ole Mors, Poorva Mudgal, Norbert Müller, Kirsten R. Müller-Vahl, Alexander Münchau, Laura Muñoz-Delgado, Tara L Murphy, Peter Nagy, Allan Naarden, Benjamin M. Neale, Muhammad S. Nawaz, Valeria Neri, Judith Becker Nissen, Markus M. Nöthen Merete Nordentoft, Ashley E. Nordsletten, Michael S. Okun, Roel A. Ophoff, Lisa Osiecki, Aarno Palotie, Teemu P. Palviainen, Peristera Paschou, Carlos N. Pato, Michele T. Pato, Christopher Pittenger, Kerstin J. Plessen, Yehuda Pollak, Cesare Porcelli, Danielle Posthuma, Eliana Ramos, Jennifer Reichert, Renata Rizzo, Mary M. Robertson, Veit Roessner, Joshua L. Roffman, Guy Rouleau, Christian Rück, Daphna Ruhrman, Evald Sæmundsen, Jack Samuels, Sven Sandin, Paul Sandor, Monika Schlögelhofer, Jeremiah M. Scharf, Simon Schmitt, Jaana Schnell, Eun-Young Shin, Paola Rosaria Silvestri, Harvey S. Singer, Liselotte Skov, Jan Smit, Jordan W. Smoller, Matthew State, Tamar Steinberg, Stian Solem, Dong-Ho Song, Jungeun Song, Sara Sopena, Hreinn Stefansson, Kári Stefansson, Nora Strom, Manfred Stuhrmann, Jin Szatkiewicz, Urszula Szymanska, Friederike Tagwerker Gloor, Zsanett Tarnok, Jay A. Tischfield, Fotis Tsetsos, Ólafur Thorarensen, Frank Visscher, Michael Wagner, Sheng Wang, Susanne Walitza, Elif Weidinger, Thomas Werge, Jeremy A. Willsey, Tomasz Wolancyk, Douglas W. Woods, Yulia Worbe, Yves Dion, Dongmei Yu, Ivette Zelaya, and Samuel H. Zinner

### EMTICS Collaborative Group

Alan Apter, Valentina Baglioni, Juliane Ball, Noa Benaroya-Milshtein, Emese Bognar, Bianka Burger, Judith Buse, Francesco Cardona, Marta Correa Vela, Andrea Dietrich, Carolin Fremer, Blanca Garcia-Delgar, Marianthi Georgitsi, Julie Hagstrøm, Tammy Hedderly, Isobel Heyman, Pieter J. Hoekstra, Chaim Huijser, Davide Martino, Marcos Madruga-Garrido, Pablo Mir, Astrid Morer, Alexander Munchau, Norbert Muller, Kirsten R. Müller-Vahl, Peter Nagy, Valeria Neri, Peristera Paschou, Kerstin J. Plessen, Cesare Porcelli, Renata Rizzo, Veit Roessner, Daphna Ruhrman, Jaana Schnell, Paola Rosaria Silvestri, Liselotte Skov, Tamar Steinberg, Friederike Tagwerker Gloor, Zsanett Tarnok, Meitar Timmor, Anne Uhlmann, Ana Vigil-Pérez, Susanne Walitza, Elif Weidinger

### TS-EUROTRAIN Network

John Alexander, Tamas Aranyi, Wim R. Buisman, Jan K. Buitelaar, Andrea Dietrich, Nicole Driessen, Petros Drineas, Siyan Fan, Natalie J. Forde, Sarah Gerasch, Odile A. van den Heuvel, Cathrine Jespersgaard, Ahmad S. Kanaan, Harald E. Möller, Muhammad S. Nawaz, Ester Nespoli, Luca Pagliaroli, Geert Poelmans, Petra J. W. Pouwels, Francesca Rizzo, Dick J. Veltman, Ysbrand D. van der Werf, Joanna Widomska, Nuno R. Zilhäo, Csaba Barta, Dorret I. Boomsma, Danielle C. Cath, Marianthi Georgitsi, Jeffrey Glennon, Bastian Hengerer, Pieter J. Hoekstra, Kirsten R. Müller-Vahl, Peristera Paschou, Hreinn Stefansson, Zeynep Tumer

### Tourette International Collaborative Genetics (TIC Genetics)

Juliane Ball, Noa Benaroya-Milshtein, Lawrence W. Brown, Keun-Ah Cheon, Barbara J. Coffey, Andrea Dietrich, Erik Elster, Dana Feldman, Thomas V. Fernandez, Carolin Fremer, Gary A. Heiman, Tammy Hedderly, Pieter J. Hoekstra, Hyun Ju Hong, Chaim Huijser, Christina Kappler Friedrichs, Robert A. King, Young Key Kim, Young-Shin Kim, Nadine Kirchen, CarolinSophie Klages, Yun-Joo Koh, Sodahm Kook, Samuel Kuperman, Bennett Leventhal, Holan Liang, Osman Malik, Pablo Mir, Astrid Morer, Marieke D. Messchendorp, Alexander Münchau, Laura Muñoz-Delgado, Tara L. Murphy, Kirsten R. Müller-Vahl, Kerstin J. Plessen, Veit Roessner, Simon Schmitt, Eun-Young Shin, Dong-Ho Song, Jungeun Song, Sara Sopena, Matthew State, Zsanett Tarnok, Jay A. Tischfield, Meitar Timmor, Anne Uhlmann, Ana Vigil-Pérez, Frank Visscher, Susanne Walitza, Sheng Wang, Jeremy A. Willsey, Samuel H. Zinner

### Tourette Association of America International Consortium of Genetics (TAAICG)

James R. Batterson, Cathy L. Barr, Cheston Berlin, Cathy L. Budman, Danielle C. Cath, Giovanni Coppola, Nancy J. Cox, Lea K. Davis, Sabrina M. Darrow, Nelson B. Freimer, Erica Greenberg, Marco Grados, Johannes Hebebrand, Matthew E. Hirschtritt, Alden Y. Huang, Joseph Jankovic, Irene A. Malaty, Carol A. Mathews, William M. McMahon, Benjamin M. Neale, Michael S. Okun, Lisa Osiecki, Danielle Posthuma, Mary M. Robertson, Guy Rouleau, Jeremiah M. Scharf, Paul Sandor, Harvey S. Singer, Jae Hoon Sul, Dongmei Yu, Gholson J. Lyon, Douglas W. Woods

### Nordic OCD and Related Disorders Consortium (NORDIC)

Julia Bäckman, Long Long Chen, James J. Crowley, Thorstein Olsen Eide, Matthew W. Halvorsen, Kristen Hagen, Jan Haavik, Bjarne Hansen, Kira D. Höffler, Fredrik Johansson, Elinor K. Karlsson, Paul Lichtenstein, Kerstin Lindblad-Toh, David Mataix-Cols, Manuel Mattheisen, Diana Pascal, Christian Rück, Elles de Schipper, Nora I. Strom, John Wallert, Gerd Kvale

## Supplementary Material

Supplementary Files

This is a list of supplementary files associated with this preprint. Click to download.

- TSCNVsupplementalmethods.docx
- TSCNVsupplementaltables.xlsx
- TSCNVsupplementalfigures.docx
